# Multiple Transitions in Permalloy Half-Ring Wires with Finite-Size Effect

**DOI:** 10.3390/ma13061384

**Published:** 2020-03-19

**Authors:** Cheng-Yi Wu, Shiow-Kang Yen

**Affiliations:** Department of Materials Engineering, National Chung Hsing University, Taichung 40227, Taiwan; skyen@dragon.nchu.edu.tw

**Keywords:** anisotropic magnetoresistances, domain wall, magnetoelectronic devices

## Abstract

Six permalloy (Py) half-rings with finite-size from 120 nm to 360 nm were connected in series on five corners. The magnetization reversal processes were investigated by the measurement of anisotropic magnetoresistance (AMR). The number of switching jumps in the AMR loops, from zero to five, varied with the longitudinal applied field. These discrete jumps resulted from domain wall (DW) nucleating and depinning on the corners. The larger external field had a fewer number of jumps in the magnetoresistance (MR) curve. This reproducible and particular response of the domain wall device in the half-ring wires pattern might be one of the new promising magnetoelectronic devices.

## 1. Introduction

Nowadays, the issue of energy consumption is crucial from every aspect. Modern electronic devices for information storage such as dynamic random access memory (DRAM) and logic processors such as metal-oxide-semiconductor field-effect transistor (MOSFET) are basically in the form of capacitors. Inevitably, capacitors must be recharged constantly to maintain their electric state, and overheating increases as size scales down. Magnetoelectronic or spin-dependent spintronic devices can offer a better solution compared with conventional semiconductor devices [1,2]. To utilize giant magnetoresistance (GMR) or tunneling magnetoresistance (TMR) effects for hard disk drives, read-head or magnetic random-access memory (MRAM) are already on the way to commercial application. However, these magnetoelectronic or spintronic devices are mainly used as storage media, and this would block developing spintronic devices. Recently, several developments of logic operation by magnetic structures, such as AND gate, NOT gate, shift register, and domain wall magnetic diodes, have been reported [3,4,5,6,7]. To fully take advantage of magnetic logic or spintronic devices, many other magnetic logic devices are wanted, such as analogue–digital converters (ADCs). More studies are also needed to measure the information carried by up or down magnetic moments and then convert it into electronic signals to be compatible with conventional electronic devices, not just by the magneto–optical signals (MOKE).

Generally, there are two kinds of magnetoelectronic devices [8]: single-domain devices, and domain wall devices [3,4,5,6,7]. Recently, interest has been on the domain wall devices for advanced lithography patterned structures to manipulate the domain wall more controllably. The half-ring or semicircular wire [9] that has better shape anisotropy in magnetic materials offers a good approach to create a domain wall pinning force by an external field. The different linewidth was used to create different magnetization states and to cause several resistance states. Therefore, we utilized this feature to design a domain wall device in a half-ring pattern with a finite-size effect. In this paper, submicron to nanometer permalloy (Ni_80_Fe_20_) half-ring in series wires with different linewidth were fabricated, and the depinning of domain walls was measured as a function of the applied field in the longitudinal direction. The number of jumps in the magnetoresistance (MR) loop decreased as the starting external fields increased. Using this relation, namely jumps versus starting external field, half-ring wires may not only act as magnetometers but also act as analogue (external field) and digital (number of jumps) converters.

## 2. Materials and Methods

**Figure 1:**
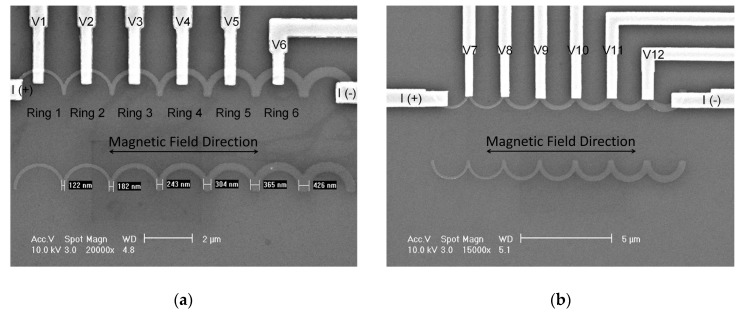
SEM micrograph of half-ring wires with I/V leads. (**a**) Middle-contact half-ring wire; (**b**) corner-contact half-ring wire.

Half-ring wires were patterned by electron-beam lithography and fabricated by the lift-off technique. Permalloy (Ni_80_Fe_20_) films, 30 nm thick, were deposited on SiO_2_/Si substrate by DC-magnetron sputtering with a collimator to reduce the side wall effect. After cleaning the surface of specimens by Ar ion-milling, the I/V (Cu (75 nm)/Au (5 nm)) contact leads were also deposited by DC-magnetron sputtering in situ. The outer diameter of each half-ring was 2 µm. The line width of half-ring corners was 120 nm to 360 nm with a step of ~60 nm. Two kinds of voltage contact layouts were designed; one was in the middle of two corners, and another was on the corners, as shown in Figure 1. We named these two half-ring wires as middle-contact half-ring and corner-contact half-ring wires in the following text. In the MR measurements, the applied current was 10 µA, which was small enough to avoid thermal activation. We also used the object-oriented micromagnetic framework (OOMMF) [10] to simulate the spin configurations of all the samples.

## 3. Results

**Figure 2:**
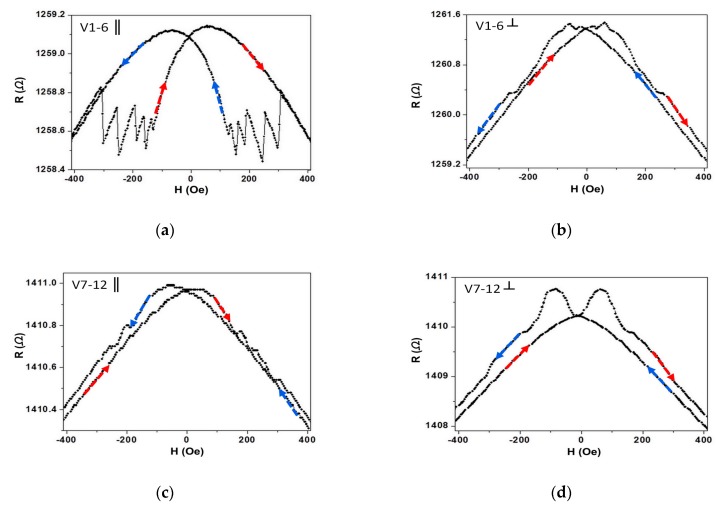
Room temperature magnetoresistance (MR) loops: (**a**) longitudinal and (**b**) transverse MR of middle-contact half-ring wire; (**c**) longitudinal and (**d**) transverse MR of corner-contact half-ring wire. The arrows indicate the direction of the proceeding process on the loop.

**Figure 3:**
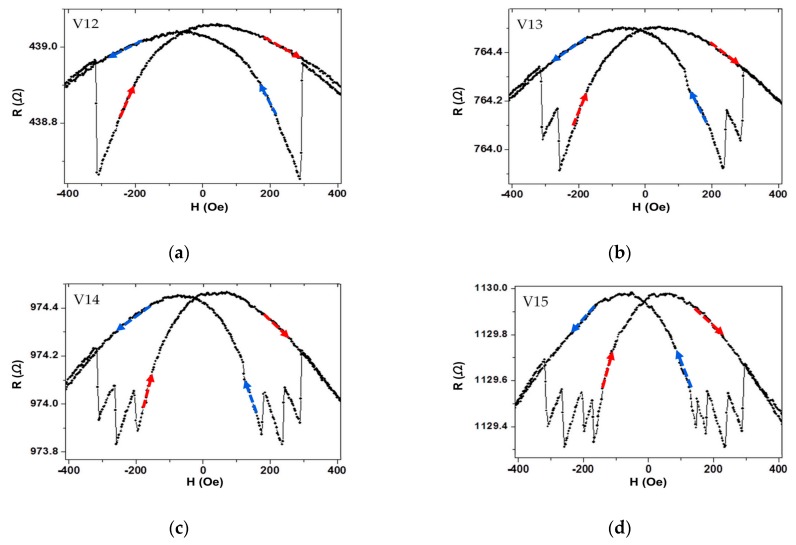
Room temperature MR loops: longitudinal MR of middle-contact half-ring wire with (**a**) voltage contact V1 and V2; (**b**) voltage contact V1 and V3; (**c**) voltage contact V1 and V4; (**d**) voltage contact V1 and V5. The arrows indicate the direction of the proceeding process on the loop.

The longitudinal and transverse MR loops of two kinds of I/V contact half-ring wires, measuring from voltage contact leads, are shown in Figure 2. As seen in Figure 2a, there were five distinct jumps in the longitudinal MR of the middle-contact half-ring wire. As seen in Figure 2c, but no distinct jump appeared in this longitudinal MR loop. As seen in Figure 2b,d, no varied jumps appeared in the transverse MR of both middle- and corner-contact half-ring wires. To investigate where these five jumps came from, we measured the MR by changing the different two voltage contacts, such as V1 and V2 (Figure 3a), V1 and V3 (Figure 3b), V1 and V4 (Figure 3c), and V1 and V5 (Figure 3d). Figure 3 shows the maximum number of jumps was equal to the corners between the voltage contact leads. By all these measurements, it was shown that the different finite-size of the half-ring wires had different Hs because of the shape anisotropy and DW pinning energy. For the discontinuity of half-ring geometry and shape anisotropy, it cost less energy to nucleate the domain wall on the corners. To reconfirm our explanation, the spin configuration of the half-ring wire was simulated by the OOMMF. The magnetic material Py parameters used for the simulations were saturation magnetization Ms = 8.0 × 10^5^ A/m and an exchange constant A = 1.3 × 10^−11^ J/m. The cell size was 5.0 nm, while the damping coefficient α was equal to 0.1. 

**Figure 4:**
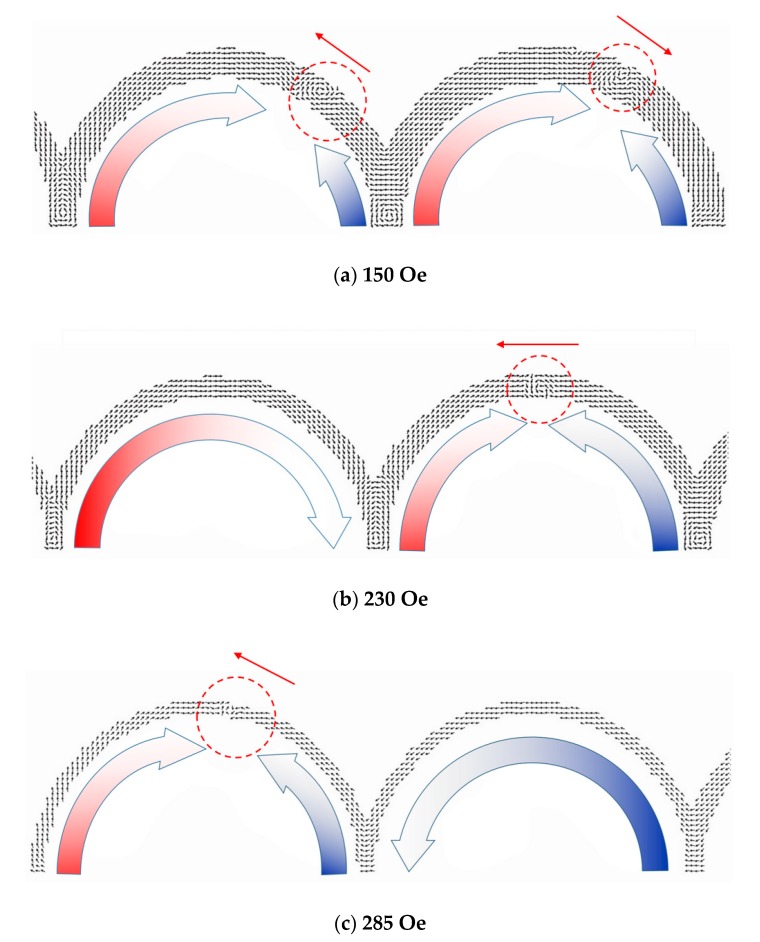
Simulation spin configuration around the switching of rings: (**a**) ring 5 and 6, (**b**) ring 3 and 4, and (**c**) ring 1 and 2. The small arrows indicate the propagation direction of domain wall (inside the dash circle), and the orientation of magnetization is illustrated by the arrow under the rings.

The first domain wall nucleation took place on the corner of the widest corner between ring 5 and ring 6. Then two domain walls propagated in ring 5 and 6 almost simultaneously to switch the magnetization from positive to negative, as shown in Figure 4a. Due to the domain walls propagating almost simultaneously, for some wider half-ring wires only four jumps and a single jump existed when the MR measurements were between voltage contacts V1 and V6, respectively. As for the other rings in the same wire, the domain walls were also nucleated on the corners but propagated only in the single-ring one from the wider corner to the thinner corner (Figure 4a–c). Accounting for the change of exchange energy versus Bx (external field), Mz/Ms (Mz: magnetization in the z axis, Ms: saturation magnetization) versus Bx and spin configuration from OOMMF simulation, the type of domain walls were all vortex domain walls from the corner width 120 nm to 360 nm. 

There were no manifest jumps in the longitudinal MR loop of the corner-contact half-ring wire as the middle-contact half-ring wire for the following reasons: first, the majority of electric current flow was in the voltage contact lead rather than the corner of the permalloy beneath, thus reducing the anisotropy magnetoresistance (AMR) contributed by the corner. Second, when the vortex wall propagated along the half-ring, the change of resistance was very little. The negative AMR ratio was proportional to the regime where local magnetization was not parallel to the current. Obviously, the magnetization transient regime of the domain wall was smaller than the corner. Third, the time for a domain wall to pass through a half-ring was less than 1 ns, calculated by a domain wall mobility of 31 ms^−1^Oe^−1^ [11], so it was not easy to detect the AMR caused by the domain wall when passing through a half-ring. Additionally, from the simulation spin configuration in the transverse field, unlike the half-ring part, the more easily magnetized axis on the corner part was parallel to the transverse direction. The magnetization reversal was dominated by the domain wall motion, and the MR change was fast and small. Thus, no manifest jump appeared in the transverse MR loops. 

## 4. Discussion

**Figure 5:**
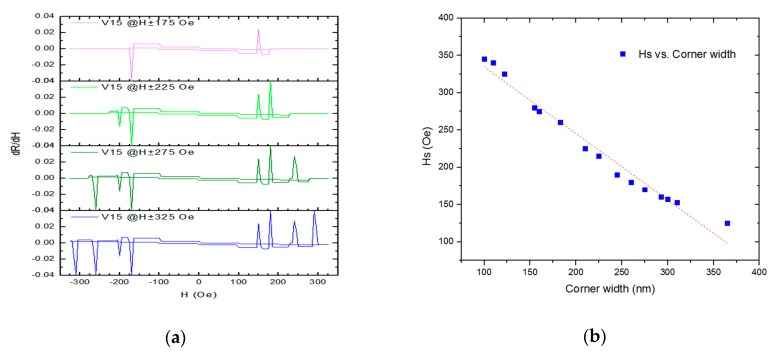
(**a**) The differential value of dR/dH versus H for the minor loop of the wider half-ring wire with a maximum of 4 jumps. (**b**) The switching field versus corner width as finite-size effect, where the dash line is the result of a linear fit.

Figure 5a shows the longitudinal minor loop of a differential MR (*d*R/*d*H) of a wider half-ring wire with voltage contact V1 and V5. The number of discrete jumps, like the digital signal, depends on the analogue signal of the external field. Therefore, the half-ring wire can act like an ADC (analogue–digital converter) by transforming the analogue signal (external field) into a digital signal (number of jumps in differential MR loop). Figure 5b shows the switching field versus line width of the corner of five half-ring wires. Between the corner width from 120 nm to 360 nm, we made a simple linear fit as a finite-size effect from these data and obtained Hs = −0.89*(corner width in nm) + 427 ± 10 (Oe). This is reasonable, as the width of the corner becomes narrow (or sharp) as the nucleation field of the domain wall become large. We may interpret the magnetization reversal process of half-ring wires as the domain wall (soliton) [3] propagates through the ring (channel or path), then traps on the corner (pinning center or energy barrier) as the external field (driving force) reaches the switching field of this corner, and the domain wall propagates again, and so on. This is compared to the magnetization reversal, which is head–head onion state to vortex state to tail–tail onion state of the micromagnet in a ring pattern [12,13]. 

The half-ring wires are six head-to-head semi-onion states in remanence of the positive saturation. As the negative field increases, a jump in the MR loop means one tail-to-tail semi-onion state increases and one head-to-head semi-onion state decreases. The corner of the half-ring plays a role as the lock or notch to reverse the onion state, and the switching field is the key to open this lock.

## 5. Conclusions

In summary, we present different linewidth in-series half-ring wires in which the switching field or domain wall depinning field depends linearly on the corner width between 120 nm to 360 nm. There are some interesting discrete jumps in the longitudinal MR of the middle-contact half-ring wire with finite-size effect. This phenomenon of the half-ring wire can be used as a micro ADC digital magnetic meter. The energy consumption by this five-bit half-ring digital micro-magnetic meter is about 1 mW (p = I^2^/R), and the reaction time is about 6 ns. We may expect that if this half-ring wire is fabricated in the spin valve structure or the domain wall is driven by spin-polarized current, the signal/noise ratio will increase, and the function will be versatile.

## References

[1] Wolf S.A., Awschalom D.D., Buhrman R.A., Daughton J.M., von Molnár S., Roukes M.L., Chtchelkanova A.Y., Treger D.M. (2001). Spintronics: A spin-based electronics vision for the future. Science.

[2] Jungwirth T., Marti X., Wadley P., Wunderlich J. (2016). Antiferromagnetic spintronics. Nat. Nanotechnol..

[3] Allwood D.A., Xiong G., Cowburn R.P. (2004). Domain wall diodes in ferromagnetic planar nanowires. Appl. Phys. Lett..

[4] Allwood D.A., Xiong G., Faulkner C.C., Atkinson D., Petit D., Cowburn R.P. (2005). Magnetic domain-wall logic. Science.

[5] Allwood D.A., Xiong G., Cowburn R.P. (2006). Magnetic domain wall serial-in parallel-out shift register. Appl. Phys. Lett..

[6] Yoshimura Y., Kim K.J., Taniguchi T., Tono T., Ueda K., Hiramatsu R., Moriyama T., Yamada K., Nakatani Y., Ono T. (2016). Soliton-like magnetic domain wall motion induced by the interfacial Dzyaloshinskii–Moriya interaction. Nat. Phys..

[7] Manipatruni S., Nikonov D.E., Young I.A. (2018). Beyond CMOS computing with spin and polarization. Nat. Phys..

[8] Cowburn R.P., Allwood D.A., Xiong G., Cooke M.D. (2002). Domain wall injection and propagation in planar Permalloy nanowires. J. Appl. Phys..

[9] Saitoh E., Miyajima H., Yamaoka T., Tatara G. (2004). Current-induced resonance and mass determination of a single magnetic domain wall. Nature.

[10] A 2-D Code to Calculate the Magnetization Configuration and Its Field Evolution Is Described on. http:/math.nist.gov/oommf.

[11] Atkinson D., Allwood D.A., Xiong G., Cooke M.D., Faulkner C.C., Cowburn R.P. (2003). Magnetic domain-wall dynamics in a submicrometre ferromagnetic structure. Nat. Mater..

[12] Kläui M., Bland C.A.F.V.J.A.C., Heyderman L.J., Nolting F., Pavlovska A., Bauer E., Cherifi S., Heun S., Locatelli A. (2004). Head-to-head domain-wall phase diagram in mesoscopic ring magnets. Appl. Phys. Lett..

[13] Sohn H., Nowakowski M.E., Liang C.Y., Hockel J.L., Wetzlar K., Keller S., McLellan B.M., Marcus M.A., Doran A., Young A. (2015). Electrically Driven Magnetic Domain Wall Rotation in Multiferroic Heterostructures to Manipulate Suspended On-Chip Magnetic Particles. ACS Nano.

